# Theta-Gel-Reinforced Hydrogel Composites for Potential Tensile Load-Bearing Soft Tissue Repair Applications

**DOI:** 10.3390/jfb14060291

**Published:** 2023-05-24

**Authors:** Charenpreet Virdi, Zufu Lu, Hala Zreiqat, Young Jung No

**Affiliations:** School of Biomedical Engineering, University of Sydney, Darlington, NSW 2006, Australia

**Keywords:** hydrogel, theta-gel, polyvinyl alcohol, soft tissue, load-bearing

## Abstract

Engineering synthetic hydrogels for the repair and augmentation of load-bearing soft tissues with simultaneously high-water content and mechanical strength is a long-standing challenge. Prior formulations to enhance the strength have involved using chemical crosslinkers where residues remain a risk for implantation or complex processes such as freeze-casting and self-assembly, requiring specialised equipment and technical expertise to manufacture reliably. In this study, we report for the first time that the tensile strength of high-water content (>60 wt.%), biocompatible polyvinyl alcohol hydrogels can exceed 1.0 MPa through a combination of facile manufacturing strategies via physical crosslinking, mechanical drawing, post-fabrication freeze drying, and deliberate hierarchical design. It is anticipated that the findings in this paper can also be used in conjunction with other strategies to enhance the mechanical properties of hydrogel platforms in the design and construction of synthetic grafts for load-bearing soft tissues.

## 1. Introduction 

The repair of high-strength, tough, and high-water content load-bearing soft tissues such as tendons, ligaments, and cartilage remains a significant challenge in the orthopaedic industry [1]. This is partly due to such tissues’ inherent poor regenerative capacity [2] and partly due to the lack of a suitable scaffold or construct that can act as scaffolds that simultaneously bear the load and allow host tissue ingrowth. Autografts and allografts are typically used in the clinic to repair such tissues [3,4]. However, autografts pose the risk of donor-site morbidity [5]. Allografts can cause undesired host-mediated immune responses, as complete decellularisation of allografts is rarely achieved to preserve the structural integrity and, thus, the mechanical properties of such allografts [6,7].

The unique combination of strength and water content in soft tissues such as tendons, ligaments, and cartilage, where the latter property is critical in the protection of the underlying tissue and distribution of forces within the collagen fibres [8,9,10], renders the engineering of synthetic materials that match such combinations difficult. In tendons and ligaments, this combination is achieved through the hierarchical structure of type I collagen microfibrils within a highly hydrated extracellular ground substance [11,12]. It is extensively documented that dry, highly aligned synthetic fibre constructs made from bioinert high-strength polymers such as ultra-high molecular weight polyethylene, polyethylene terephthalate, and polytetrafluoroethylene are subject to foreign body responses characterised by the presence of multinucleated giant cells that eventually act to degrade the fibrous constructs, rendering them mechanically weaker and substantially increasing the risk of rupture around the ~5–15 years post-implantation period [13,14,15,16,17].

A promising approach moving forward with the engineering of synthetic constructs for load-bearing soft tissues is enhancing the mechanical strength of existing hydrogel materials without using such high-strength, bioinert polymeric fibres. Hydrogels provide a promising option for soft tissue repair due to their ability to facilitate biomolecule conjugation, cell seeding, and tissue ingrowth due to the inherent microporous structures and high-equilibrium water content, which enables the transfer of nutrients and wastes within the hydrogel construct [18,19,20,21,22,23]. Various manufacturing techniques, such as mould casting and additive manufacturing, may also be employed to shape hydrogel constructs [24,25,26]. The key limitation for hydrogels, either for synthetic hydrogels such as polyvinyl alcohol (PVA) and polyacrylamide (PAAm) or naturally-derived hydrogels such as chitosan and alginate, as standalone unmodified materials is their lack of initial mechanical strength and stiffness to enable its use as a temporary tendon and ligament graft whilst allowing for the slow regenerative processes to take place. 

For this study, we investigate the mechanical modification of polyvinyl alcohol hydrogels formed through the theta-gelation method (called ‘theta gels’) [27]. Theta-gelation is a mechanism whereby a phase separation induced through the addition of a low-molecular-weight polymer—typically polyethylene glycol—into a solution of high-molecular-weight PVA at high temperatures around 70 to 90 °C. Upon cooling, the solution undergoes a thermal phase transition—this results in dense PVA regions and subsequent induced crystallization via physical crosslinks [28,29]. Due to its thermoreversible property arising from reversible physical crosslinks, PVA theta gels possess the unique property of facile manufacturing of various structures through mould casting using basic heating equipment and cooling at ambient conditions or refrigeration [27]. PVA theta gels have subsequently been reported to have shown excellent toughness and creep resistance compared to other existing hydrogels [30]. These theta gels can readily incorporate soluble biological molecules such as gelatine [28,31] and synthetic bioactive ceramics [32] to alter their biological properties. Notably, theta gels do not employ the use of potentially toxic chemical crosslinkers such as glutaraldehyde to achieve moderate hydrogel strengths [33,34,35,36]. We have also previously shown that this material, in the presence of aligned ultra-high molecular weight polyethylene fibres and gelatin integration, supports the ingrowth of collagenous tissue into its structure when implanted into rat patellar tendon defects [32].

Due to such ideal properties of PVA theta gels of non-toxicity, biocompatibility, relatively high strength versus other polymer-based hydrogels, and ease of manufacturing, we have selected this hydrogel for this study to demonstrate a proof-of-principle method to mechanically draw the PVA theta gels to form high-strength theta gel toric bands. We then use these drawn PVA theta gel toric bands to engineer a ‘theta-gel-reinforced hydrogel’ constructs where we form bundles of drawn theta-gel toric bands at various densities and then inject PVA hydrogels within and around pre-drawn theta-gel bundles—which we hypothesise would substantially increase the hydrogel’s tensile properties. This study characterises the change in mechanical properties and microstructure, as well as its impact on equilibrium water content that is known to influence key biological properties such as tissue ingrowth and nutrient-waste transfer [18,20,37].

## 2. Materials and Method

### 2.1. Preparation of Toric PVA Theta Gels

The polyvinyl alcohol (PVA) theta gels were prepared using a method adapted from previous studies [27,32]. Briefly, an aqueous solution of dissolved 10 wt.% PVA (M_w_ = 89,000–98,000, >99% hydrolysed; Sigma Aldrich, Burlington, MA, USA), 1 wt.% gelatin (bovine skin type B; Sigma Aldrich, USA), and polyethylene glycol M_n_ = 400 (PEG; Sigma Aldrich, USA) were homogenously mixed at ~90 °C and vortex at a ratio of 72:28 wt.%.

**Figure 1:**
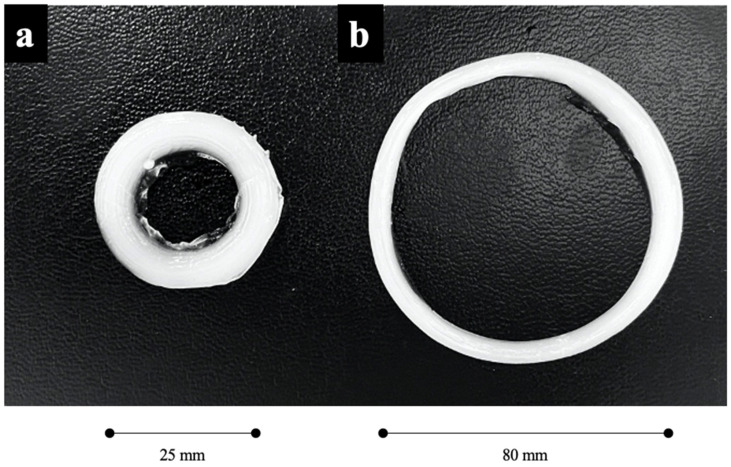
Photograph of the PVA toric theta gels. (**a**) Undrawn (DR−); (**b**) Drawn (DR+).

To form the PVA theta gels tori, the mixed PVA-PEG solution was dispensed into a torus-shaped mould with dimensions of 5 mm in cross-sectional diameter and an average torus diameter of 25 mm. The solution was then left to gel at ambient room temperature (25 °C) for 10 min and removed from the mould (Figure 1a). After 10 min of gelation, a subset of the PVA hydrogel tori was mechanically drawn until the new torus diameter reached 80 mm (Figure 1b). This was the maximum diameter reliably synthesised through this method without introducing induced cracks into the toric theta-gel structure, resulting in an average cross-sectional diameter of ~2 mm in the drawn theta-gel torus. The drawn (DR+) and undrawn (DR-) theta gel tori were then stored in deionised water overnight to remove the PEG from the DR theta gel. 

The impact of a single round of freeze drying the PVA theta gels on its mechanical properties was also investigated. DR+ and DR- PVA theta-gel tori were removed from their stored solution and placed in the −80 °C freezer for at least 24 h. The samples were then subsequently freeze-dried overnight (Labconco, Kansas City, MO). The freeze-dried (FD+) tori were rehydrated for characterisation; a subset of the theta-gel tori was not subjected to the freeze-drying process (FD−). This resulted in four groups of PVA theta-gel tori synthesised: drawn and freeze-dried (DR+/FD+), drawn and non-freeze-dried (DR+/FD−), undrawn and freeze-dried (DR−/FD+), and undrawn and non-freeze-dried (DR−/FD−).

### 2.2. Crystallinity and Microstructure

The effect of drawing on the crystallinity, melting temperature, and microstructure of the toric theta gels was investigated on the DR+/FD+ and DR−/FD+ samples. Differential scanning calorimetry (DSC) (TA Instruments, DSC Mod. 2920) was carried out with a heating rate of 10 °C/min up to 260 °C on the DR+/FD+ and DR−/FD+ toric hydrogels to observe differences in the crystallinity between the two groups. The microstructure of the surface topography of DR+/FD+ and DR−/FD+ toric hydrogels was examined under scanning electron microscopy (SEM, TM3030Plus, Hitachi, Tokyo, Japan). The samples were cut and sputter coated with gold before the microstructural examination. Non-freeze-dried groups (i.e., DR+/FD− and DR−/FR−) could not be characterised due to the need for the samples to be dehydrated for reliable characterisation of crystallinity and microstructure.

### 2.3. Synthesis of Hydrogel-Reinforced Theta-Gel Tori 

**Figure 2:**
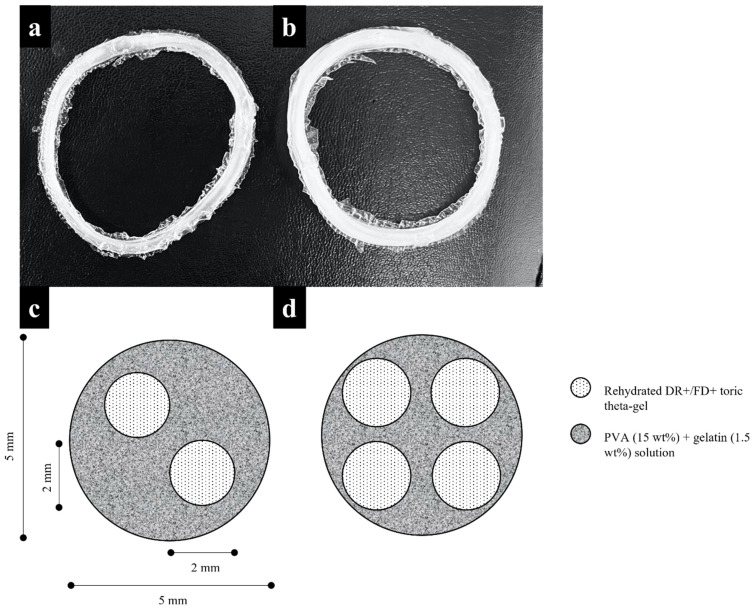
(**a**,**b**) Photograph and (**c**,**d**) accompanying schematic of the cross-section of the freeze-dried theta-gel-reinforced hydrogels. (**a**,**c**) LD-TRH; (**b**,**d**) HD-TRH.

DR+/FD+ tori were subsequently used to produce low-density theta gel-reinforced hydrogels (LD-TRH) and high-density theta-gel-reinforced hydrogels (HD-TRH) for mechanical characterisation. The rehydrated DR+/FD+ toric theta-gel samples were placed in toric moulds of 80 mm diameter and 5 mm cross-sectional diameter—two toric theta gels were used for LD-TRH (Figure 2a,c). Four toric theta gels were used for HD-TRH (Figure 2b,d). The moulds were then filled with an aqueous solution of PVA (15 wt.%) and gelatin (1.5 wt%) and then subjected to six rounds of freeze-thawing, with each freezing and thawing stage carried out overnight. After the sixth freeze–thaw round, the LD-TRH and HD-TRH samples were rehydrated with deionised water.

### 2.4. Equilibrium Water Content

The equilibrium water content (EWC) for four toric theta-gel groups DR+/FD+, DR−/FD+, DR+/FD−, and DR−/FD−, and the two hydrogel-reinforced theta-gel groups LD-HRT and HD-HRT. For the DR+/FD− and DR−/FD− groups, the EWC was calculated as follows: [(weight of hydrated theta-gel—the weight of freeze-dried theta-gel)/(weight of hydrated theta-gel)] × 100%; for the DR+/FD+, DR−/FD+, LD-HRT and HD-HRT groups: [(weight of rehydrated theta-gel—the weight of freeze-dried theta-gel)/(weight of rehydrated theta-gel)] × 100%, where the rehydration involved immersing the samples in deionised water overnight in a 37 °C incubator after the freeze-dry treatment. N = 4 samples for each group were examined.

### 2.5. Tensile Mechanical Properties

Ultimate tensile strength, tensile modulus, and tensile strain at failure were obtained by subjecting the toric samples of DR+/FD+, DR−/FD+, DR+/FD−, and DR-/FD−, and the two hydrogel-reinforced theta-gel groups LD-HRT and HD-HRT. All groups were rehydrated in deionised water for at least 12 h at 37 °C before mechanical testing. The toric samples were hooked on two ends of the universal testing machine (Instron 5567, Bluehill, Norwood, MA, USA). All samples had a pre-load of 0.1 N before testing and were subject to a tensile ramp rate of 15 mm/min, using a 1 kN load cell. The tensile strength and modulus of the specimens were then obtained according to ASTM D3039 standards.

### 2.6. Statistical Analysis

All data are presented as mean ± standard deviation (SD). For statistical analysis, Levene’s test was performed to determine the homogeneity of variance of the data, and then either Tukey’s honestly significant difference or Tamhane’s post hoc tests were used. A statistics programme (SPSS, Chicago, IL, USA) was employed for all statistical analyses and differences were considered statistically significant if the obtained *p*-value was <0.05.

## 3. Results

### 3.1. Crystallinity and Melting Temperature of Drawn and Undrawn Theta Gels

The mean crystallinity for the drawn DR+/FD+ PVA theta gels was 54.5 ± 0.4% and was found to be significantly higher (*p* < 0.05) than that of the undrawn DR−/FD+ PVA theta gels (46.1 ± 1.5%). The mean melting temperature for the DR+/FD+ PVA theta gels was 232.3 ± 0.1 °C versus that for the DR-/FD+ PVA theta gels at 230.9 ± 0.1 °C.

### 3.2. Microstructure

**Figure 3:**
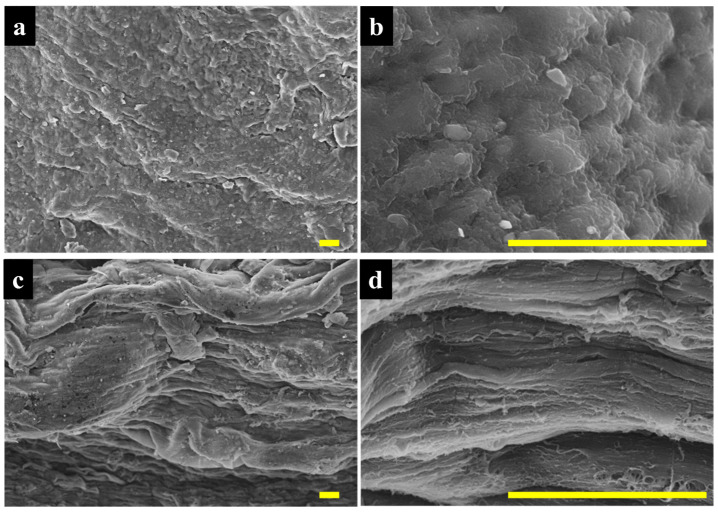
Scanning electron micrographs of the PVA theta gels; (**a**,**b**) DR−/FD+; (**c**,**d**) DR+/FD+. The scale bar represents 20 µm.

Figure 3a,b shows the surface microstructure of the DR−/FD+ PVA theta-gel samples at 500× (Figure 3a) and 5000× magnification (Figure 3b). The microstructure of DR−/FD+ theta-gel samples showed a relatively homogenous microstructure typically observed in dry hydrogels with closed pores. In contrast, we observed a highly longitudinally oriented microstructure along the direction of mechanical drawing for the DR+/FD+ PVA theta-gel samples at 500× and 5000× magnification (Figure 3c,d, respectively). 

### 3.3. Equilibrium Water Content

**Figure 4:**
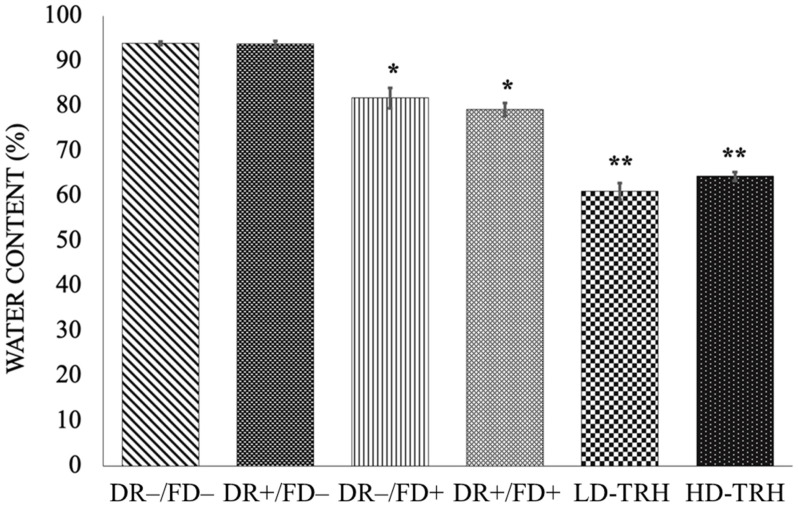
Equilibrium water content exhibited by the toric theta gels and theta-gel-reinforced hydrogels. *: *p* < 0.05 vs. DR−/FD− and DR+/FD−; **: *p* < 0.05 vs. DR−/FD−, DR+/FD−, DR−/FD+, and DR+/FD+.

The non-freeze-dried theta-gel samples showed the highest equilibrium water content compared to the freeze-dried theta-gel samples and the theta-gel-reinforced hydrogels (Figure 4). DR−/FD− and DR+/FD− theta gels showed EWC of 94.0 ± 0.4% and 93.9 ± 0.6%, respectively. These EWC values were statistically significantly higher than the freeze-dried theta-gel counterparts—DR−/FD+ and DR+/FD+ theta gels exhibited EWC of 81.8 ± 2.3% and 79.3 ± 1.5%, respectively. No statistically significant difference in the EWC between the drawn and undrawn samples for freeze-dried (FD+) and non-freeze-dried groups (FD−). The theta-gel-reinforced hydrogels exhibited the lowest EWC, with LD-TRH and HD-TRH exhibiting EWC of 61.0 ± 1.9% and 64.4 ± 1.0%, respectively, with no significant difference between LD-TRH and HD-TRH.

### 3.4. Tensile Mechanical Properties

**Figure 5:**
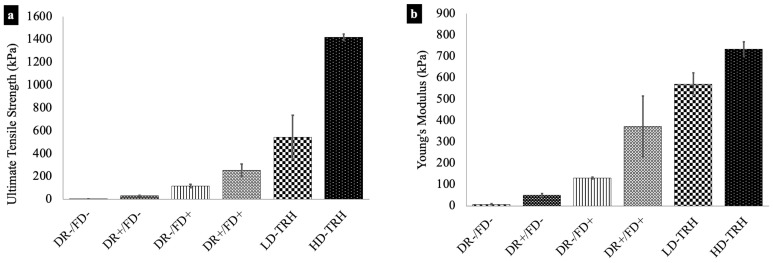
(**a**) Ultimate tensile strength and (**b**) tensile modulus of the theta gels and theta-reinforced hydrogels. All groups are statistically significantly different from each other (*p* < 0.05).

The tensile strength and modulus of the constructed theta gels subject to either mechanical drawing or freeze-dry regime, as well as the theta-gel-reinforced hydrogels, are reported in Figure 5. The non-freeze-dried PVA theta gels showed the lowest tensile strength and modulus, with DR−/FD− theta gels exhibiting 2.3 ± 1.9 kPa and 7.4 ± 3.9 kPa, respectively, and DR+/FD− theta gels exhibiting 31.4 ± 5.1 kPa and 50.0 ± 7.9 kPa, respectively. A statistically significant increase (*p* < 0.05) was observed for the drawn DR+/FD− theta gels versus the undrawn DR−/FD− theta gels.

The freeze-dried theta gels exhibited significantly higher than their non-freeze-dried counterparts (*p* < 0.05). DR−/FD+ theta gels showed tensile strength and modulus of 117.1 ± 14.8 kPa and 130.8 ± 4.8 kPa, respectively, and the DR+/FD+ theta gels exhibited tensile strength and modulus of 254.2 ± 55.0 kPa and 371.5 ± 142.6 kPa, respectively. The drawn freeze-dried theta gels showed higher tensile mechanical properties than the undrawn freeze-dried theta gels.

The theta-gel-reinforced hydrogels exhibited the highest tensile strengths and modulus. LD-TRH showed tensile strength and modulus of 545.4 ± 190.2 kPa and 570.3 ± 52.6 kPa, respectively. 

HD-TRH showed tensile strength and modulus of 1417.8 ± 190.2 kPa and 570.3 ± 52.6 kPa, respectively. The increase in both tensile strength and modulus for HD-TRH when compared to LD-TRH is statistically significant (*p* < 0.05).

## 4. Discussion

This study has demonstrated for the first time that synthetic PVA hydrogels using purely physical crosslinking methods and without potentially toxic chemical crosslinkers such as glutaraldehyde [34] have achieved tensile strengths in the MPa range for the HD-TRH group through mechanical drawing, freeze-drying, and structured reinforcement. All reagents used in the engineering and synthesis of the theta gels used in this study, i.e., PVA and PEG, have long been considered biocompatible within the human body [38]. This study also provides a facile framework to manufacture hydrogel-reinforced hydrogels with hierarchical structures to achieve increased tensile and mechanical properties whilst retaining the high-equilibrium water content similar to those seen in native human tissues. Studies where hydrogel strengths exceeded 1 MPa have used either chemical crosslinking [39,40,41] or other more complex methods such as freeze-casting [42], freeze-assisted salt-leaching [42], or molecular self-assembly [43]. This study would be useful as a reference for future studies wanting to engineer strong hydrogel constructs to repair or replace soft tissues in humans. It is foreseeable that the methods to increase the mechanical properties of synthetic hydrogels, such as those made from PVA presented in this study, could be synchronised with other production methods to meet the design needs of a given application.

The choice of the theta-gelation method of PVA hydrogels [27] to generate the toric theta gels and hydrogel-reinforced theta-gel samples used in this study is deliberate. Firstly, the use of self-setting PVA theta gels enables the synthesis of consistent samples using moulds similar to those used in injection moulding and enables reliable physical removal from such moulds after partial setting, where the degree of setting can be essentially controlled by controlling the time for the gel to set and the ambient temperature. This partially set stage of the theta gel enables the mechanical drawing to align the hydrogel’s porous microstructure in its principal direction, subsequently increasing its tensile strength along such a given direction. Increasing the diameter of the torus of the partially set hydrogel in a controlled mechanical manner forces the hydrogel mesh and crystalline structure to align. This drawing process is not possible with the conventional freeze-thawing gelation methods typically used in PVA hydrogel synthesis [44], nor any chemical crosslinking mechanisms [45], as the gelation process is required to be taken through to completion within the vessel that encases the initial PVA solution before reliable removal and transfer due to the nature of such methods.

The toric shape of high-strength hydrogels is also essential for the reliable characterisation of tensile mechanical properties. Often, pneumatic grips are used to fix hydrogel samples in place for tensile property characterisation; this results in two undesired effects that decrease the reliability of such results—firstly, the hydrogel around the grips is heavily deformed due to the highly compliant nature of hydrogels. Thus, the hydrogels tend to break prematurely at the grips. Secondly, due to the hydrated nature of the hydrogels, the surface film of water reduces the required friction and often results in slippage in the grips. The toric samples enable reliable tensile characterisation of hydrogels as hooks rather than grips can be used to ensure entirely axial loading of the hydrogel samples.

With the mechanical drawing of the theta gels, we see a substantial increase in crystallinity and melting temperature, indicative of the increased alignment of the molecular structure of the DR+ hydrogels, and we were successfully able to induce this change by mechanically drawing partially set theta gels. Mechanical drawing is a widely used method to increase crystallinity and tensile mechanical strength along the principal direction of drawing in semicrystalline polymers. Drawing is also the foundation of spinning techniques to produce dry, high-strength synthetic fibres, and physical processes such as melt extrusion [46] and electrospinning [47] can be employed to generate fibres of diameter < 100 microns. However, at this stage, reliably generating ‘hydrogel fibres’ with high EWC using purely physical gelation methods similar to melt extrusion is challenging due to the low viscosity of the intermediate setting hydrogel solution. Even in this study, we found that removing the theta-gel samples from their moulds too early during the partial setting results in uncontrolled ‘flow’ of the theta gel; conversely, removing the theta-gel pieces from their moulds too late renders the hydrogel unable to be drawn mechanically. During our optimisation processes, we found that removing the partially setting hydrogel samples 10 min from initial heating and injection leads to reliable samples and enables sufficient mechanical drawing.

This study makes it evident that the mechanical drawing of the theta gels alone does not reduce the EWC of the theta gels, regardless if they have been treated with the freeze-drying process, and that the improvements in the tensile mechanical properties we observed for the DR+ theta gels do not arise from a loss in EWC, but rather the longitudinal alignment of the drawn hydrogel itself, and to a certain extent, the increase in crystallinity. This observation implies that the mechanical strengths of hydrogels can be enhanced without necessarily reducing the EWC of the hydrogel itself. The microstructure also indicates that the mechanical drawing process is successful and manifests at the surface microstructure, as indicated by the collapsed pores’ alignment in Figure 3. It is important to note that aligned microstructures, both structurally, i.e., fibrous, and at the topographical level, are critical in influencing cell behaviour, particularly the tenogenic cells such as tenocytes and tenoblasts [1]. Previous studies on PVA theta gels have shown good biocompatibility in in vitro [28] and in in vivo settings for both cartilage [48] and tendon [32]. Future studies on this material will aim to explore the influence of such topography on tenogenic cells; preliminary studies have confirmed that the theta gels used in this study do indeed support the viability of murine osteoblasts (Supplementary Figure S1). The influence of the microstructure of these constructs on cell behaviour remains to be investigated—it is well-established, however, that the surface topography and alignment of surface features play an influential role in the biological behaviour of resident cells. PVA hydrogels without additional functionalization of the PVA backbone, such as the material system employed in this study, is considered non-degradable [44,48], though PVA hydrogel systems such as tyramine functionalisation can be modified to support biodegradation [31]. 

The freeze-drying process is necessary for longer-term storage and enabling off-the-shelf availability of such materials. It is also known to increase crystallinity substantially [49] by forcing the crystalline regions together, subsequently increasing the mechanical strength and decreasing the hydrogel’s equilibrium water content. This study showed that the freeze-drying process increased the tensile strength and modulus of the rehydrated theta gels, resulting in a loss of equilibrium water content. A high EWC whilst retaining the mechanical strength is important in the engineering of synthetic hydrogels for soft tissue repair, as they are critical for maintaining lubrication [50], transport of nutrients within the synthetic graft through the pores, and act as a ‘cushioning mechanism’ to protect the physical mesh structure. Soft load-bearing tissues such as cartilage, ligaments, and tendons exhibit an EWC of 60–80% [1,51]. 

To further improve the strength of the engineered hydrogel constructs into the MPa range, we reinforced a conventional PVA hydrogel with the toric theta gels synthesised in this study, acting similarly to fibres of fibre-reinforced composites. The LD-TRH and HD-TRH composites demonstrated that hydrogels could be further reinforced with aligned hydrogels to substantially enhance the mechanical properties in the direction of the alignment. Furthermore, it is anticipated that the porous nature of the reinforcement hydrogels would enable a strong interface between the matrix hydrogel and the dispersed hydrogel bands within the LD-TRH and HD-TRH as the matrix hydrogel would be made to crosslink around the dispersed hydrogel bands.

In summary, this study demonstrated that biocompatible hydrogel constructs made from PVA-based materials with high-equilibrium water content (>60%) could be made to reach >1 MPa in tensile strength through facile manufacturing methods and via physical crosslinking without the use of chemical crosslinkers nor use of complex processes such as freeze-casting. Future studies on this material system would look to generate highly elongated theta gels and increase the density of the aligned theta gels within the theta-gel-reinforced hydrogel systems to further increase the mechanical properties without compromising the biocompatibility, equilibrium water content, and the facileness of the manufacturing processes.

## Data Availability

The data presented in this study are available on request from the corresponding author.

## Supplementary Materials

The following supporting information can be downloaded at: https://www.mdpi.com/article/10.3390/jfb14060291/s1, Figure S1: Live cell staining of murine fibroblasts after 7 days of culture on (a) DR−/FD+ and (b) DR+/FD+ theta-gels.
