# Supplementary material for: Theta-Gel-Reinforced Hydrogel Composites for Potential Tensile Load-Bearing Soft Tissue Repair Applications
Source: J Funct Biomater. 2023 May 24;14(6):291. doi: 10.3390/jfb14060291 (PMC10299711; doi:10.3390/jfb14060291)
Supplement: Supplementary file 1 [file jfb-14-00291-s001.zip › jfb-2401626-supplementary.pdf]

SUPPLEMENTARY DOCUMENTS

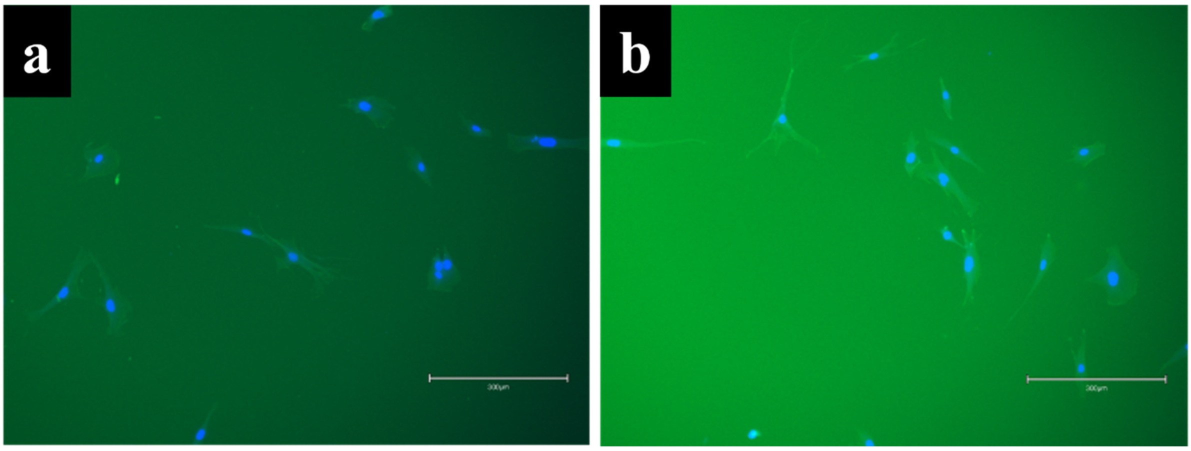

Figure S1. Live cell staining of murine fibroblasts after 7 days of culture on (a) DR-/FD+ and (b) DR+/FD+ theta-gels.
